# Epidemiological trends of diarrheal viruses in central and western Kenya before and after Rotavirus vaccine introduction

**DOI:** 10.1186/s41182-025-00716-6

**Published:** 2025-04-27

**Authors:** Maurine Mumo Mutua, Cyrus Kathiiko, Mary N. Wachira, Betty Muriithi, James Nyangao, Samoel A. Khamadi, Satoshi Komoto, Kouichi Morita, Yoshio Ichinose, Ernest A. Wandera

**Affiliations:** 1 Institute of Tropical Medicine, Nagasaki University-Kenya Medical Research Institute, Nairobi, Kenya; 2 KEMRI Graduate School of Health, Nairobi, Kenya; 3 Centre for Virus Research, Kenya Medical Research Institute, Nairobi, Kenya; 4 Division of One Health, Research Center for Global and Local Infectious Diseases, Oita University, Oita, Japan

**Keywords:** Adenovirus, Norovirus, Astrovirus, Rotavirus-vaccine, Kenya

## Abstract

**Background:**

Rotavirus, norovirus, adenovirus (type 40/41) and astrovirus are the most significant viral etiological agents of acute gastroenteritis in young children globally. Kenya introduced the rotavirus vaccine into her National Immunization Program in July 2014, which has led to a significant decline in the prevalence of rotavirus. We sought to assess the impact of rotavirus vaccination on the epidemiological trends of other diarrhea-associated enteric viruses across different regions in Kenya.

**Methodology:**

Using conventional and multiplex RT-PCR, we analyzed a total of 716 fecal samples for adenovirus, astrovirus and norovirus from children aged below 5 years presenting with acute gastroenteritis but tested negative for rotavirus at Mbita Sub-County Referral Hospital in Western Kenya and Kiambu County Referral Hospital in Central Kenya before (2011–2013) and after (2019–2020) rotavirus vaccine introduction.

**Results:**

Following the rotavirus vaccine introduction, there was no significant difference in norovirus and astrovirus prevalence post-vaccine introduction in both Central (norovirus- 5.4% vs 5.9%; astrovirus- 2% vs 2.4%) and Western Kenya (norovirus- 2% vs 3%; astrovirus 3.3% vs 5.9%). Although the prevalence of adenovirus increased substantially in Western Kenya (9% vs 12.4%), there was a significant decrease in adenovirus in Central Kenya (17%, vs 6%, *p* = 0.007).

Before the introduction of the rotavirus vaccine, a large proportion of adenovirus cases occurred at 6–8 months in Central Kenya and 12–23 months in Western Kenya, while norovirus prevalence was highest at 12–23 months in Central and 3–5 months in Western Kenya. Astrovirus infections in Central Kenya were predominantly among children aged 12–23 months, both before and after the vaccine. Following vaccine introduction, a large proportion of adenovirus cases occurred among children aged 12–23 months in both regions. Norovirus peaked at 12–23 months in Central Kenya and showed dual peaks at 3–5 and 9–11 months in Western Kenya. Astrovirus infections in Western Kenya shifted from peaks at 6–8 and 24–59 months pre-vaccine to 9–11 months post-vaccine.

**Conclusion:**

Our data demonstrate the burden and changing epidemiology of enteric viruses in Western and Central Kenya and underscores the need for continued monitoring to guide the design and implementation of appropriate public health interventions.

## Background

Diarrheal viruses such as human astrovirus, norovirus, adenovirus (type 40/41), and rotavirus, are a significant contributor to acute gastroenteritis (AGE) globally [1] and a leading cause of illness and death among children under five in low and middle-income countries (LMICs) [2]. Diarrhea is the third cause of mortality in children below 5 years globally, with viral diarrhea accounting for the most causative pathogen and LMICs reporting over 3000 mortality cases due to AGE [1, 3] Enteric viruses infect the mammalian gastrointestinal tract [4] and the clinical symptoms manifest as fever, malaise, vomiting, and diarrhea [5–7]. Before the vaccine introduction, rotavirus was the leading cause of viral AGE globally [8, 9].

Two rotavirus vaccines were approved and licensed in 2006 [10] by the World Health Organization (WHO) for rotavirus prevention, with Kenya introducing Rotarix (GlaxoSmithKline, Rixensart, Belgium), a monovalent vaccine into the National immunization program in July 2014 [11]. The introduction of rotavirus vaccine has led to a significant decrease in AGE globally [12] with developed countries reporting higher impact and effectiveness [13] than low and middle-income countries [14] Despite the low prevalence of other enteric viruses before vaccine introduction, there have been concerns about their incline, to replace rotavirus following vaccine introduction. For instance, some studies have shown norovirus being the leading cause of AGE replacing rotavirus following rotavirus vaccine introduction [6, 15].

Norovirus is a non-enveloped RNA virus of the *Caliviridae* family causing morbidity across both community and healthcare settings [16]. It has high mutation rates and is classified into 10 genogroups and 49 genotypes [17]. GI and GII are predominant in causing gastroenteritis, with GII being responsible for the clinical manifestation [15, 16]. Human astroviruses are positive-sense single-stranded RNA viruses of the *Astroviridae* family [18]. They have a known etiology of 2–8% of AGE cases in Africa [19]. Human adenoviruses are double-stranded DNA viruses belonging to the *Adenoviridae* family [20] that typically cause mild infections [7]. Adenovirus 40/41 have a high affinity for the gastrointestinal tract leading to childhood diarrhea [21].

There is limited data globally on the impact of rotavirus vaccination on the epidemiological trends of other diarrhea-associated enteric viruses such as norovirus, adenovirus (type 40/41), and astrovirus. While the burden and molecular epidemiology of rotavirus before and after vaccine introduction are well described globally, these data are lacking for norovirus, adenovirus 40/41 and astrovirus in Kenya and other developing countries. Furthermore, with the reported decline of rotavirus disease after vaccine implementation, monitoring for other diarrheal viruses will be necessary to assess changes in their relative burden following rotavirus vaccine introduction and implement appropriate public health measures. In this study, we aimed to determine the disease burden and molecular epidemiology of norovirus, adenovirus 40/41 and astrovirus among rotavirus negative children in Central and Western Kenya before and after rotavirus vaccine implementation. Understanding the impact of rotavirus vaccination on the epidemiological patterns of these viruses is essential for designing and prioritizing interventions.

## Methods

### Study design and setting

We conducted a hospital-based retrospective cross-sectional surveillance, using archived rotavirus-negative fecal samples obtained from children below 5 years presenting with acute gastroenteritis (AGE) between June 2011 and January 2014 (hereby referred to as the pre-rotavirus vaccine period) and between February 2019 and October 2020 (referred to as the post-rotavirus vaccine period) at Kiambu County Referral Hospital (KCRH) in Central Kenya and Mbita Sub-County Referral Hospital (MSRH) in Western Kenya. The participants presented with at least three episodes of looser-than-normal or watery stools in 24 h for not more than 7 days with or without episodes of vomiting. The children either came from the community or were referred to by peripheral hospitals. Both KCRH and MSRH are among the largest referral hospitals in the respective regions of Kenya and serve both inpatient and outpatient populations.

### Sample collection

Clinical diagnosis of the children was conducted by the attending clinicians in both central and western Kenya to determine their eligibility for inclusion in the study. After obtaining parental informed consent, demographic and clinical data about the eligible children were recorded and fecal samples were collected in clean sterile containers. Each sample was labeled according to the date of collection and the sample number. The samples were kept at 4 °C at the hospital before being transported to the Nagasaki University, Institute of Tropical Medicine-Kenya Medical Research Institute where they were stored at − 80 °C until processing.

### Sample processing

1 ml of a 10% fecal suspension was prepared from 100 µl of a rectal swab suspension in 0.01 M phosphate-buffered saline (PBS) (pH 7.2) as described previously [22]. All samples were subjected to RNA extraction for astrovirus and norovirus, using TRizol reagent as per manufacturer's protocol and DNA extraction for adenovirus, using Quick-DNA™ Fecal/Soil Microbe Mini-prep Kit (Zymo Research, UK) according to manufacturers' protocol. cDNA synthesis and multiplex conventional PCR were done using a One-Taq-One-Step RT-PCR kit (BioLabs, England) according to the manufacturer’s protocol under the following conditions; 48 °C for 45 min, 94 °C for 2 min, 35 cycles (95 °C for 30 secs, 53 °C for 1 min and 72 °C for 2 min), 72 °C for 7 min and 4 °C ∞, using Mon 340 (Forward primer 5′-CGTCATTGTTTGTTGTCATACT-3′) and Astman-2 (reverse primer 5′-TCGCTTCATACATCAAACCC-3′) primer set for astrovirus and G1 (Forward primer 5′-TATGGTGATGATGAAATAGTGTC-3′ and reverse primer 5′- ATTTCGGGCAGAAGATTG-3′) and G11 (Forward primer 5′-GCACACTGTGTTACACTTCC-3′ and reverse primer 5′- ACATTGGCTCTTGTCTGG-3′) for norovirus. Adenovirus40/41 (Forward primer 5′-GCCACCGATACGTACTTCAGCCTG-3′ and reverse primer 5′-GGCAGTGCCGGA GTAGGGTTTAAA-3′) was screened by conventional PCR using puReTaq Ready-To-Go™ PCR Beads according to the manufacturer's protocol under the following conditions; 94 °C for 2 min, 35 cycles (94 °C for 30 secs, 59 °C for 1 min and 72 °C for 1 min), 72 °C for 7 min and 4 °C ∞,. The amplified products were analyzed using 1.5% agarose gel electrophoresis.

### Data extraction and analysis

Adenovirus, astrovirus, and norovirus prevalence before and after rotavirus vaccination was determined by dividing the positive samples over the total samples for each study site and period and expressed as percentages. The age demographic distribution of the viruses during the study period was determined. All data analyses were done using EPI Info version 3.5.3.2. Differences in proportions were tested using the CI, mid-P exact. Additionally, we obtained card-confirmed administrative data on rotavirus vaccinations from each of the child enrolled in the study. Using these data, we estimated the percentage of rotavirus vaccination coverage among children infected with adenovirus, astrovirus, and norovirus by dividing the number of children positive for each of the viruses and vaccinated with rotavirus (numerator) by the total number of children positive for each of the viruses (denominator) in each of the study sites. Diarrheal disease severity was assessed using the 20-point Vesikari Clinical Severity Scoring System (VCSS) [23]. The seven scoring parameters—diarrhea, vomiting, fever, dehydration, and the duration of diarrhea and vomiting and treatment status—were included in the VCSS. Since the total duration of each episode and the symptom severity from the first day of an episode should be considered, symptoms were collected retrospectively from each child from the day that any single symptom (i.e., diarrhea and/or vomiting) began rather than from the day of presentation to the health facility. The scores for each parameter within the clinical severity scoring system were added allowing for a severity score between 0 and 20 points. Severity scores above 10 points (i.e., ≥ 11 points) were considered severe; scores between 7 and 10 were moderate; and scores less than 7 were mild.

## Results

### Demographic characteristics of the study population

In the pre-vaccine era in Western Kenya, the largest proportion of children presenting with AGE was among 24–59 months (34%) age category. Following vaccine introduction, the largest proportion of AGE was among children in the 12–23 age category (29.4%). In central Kenya, AGE was observed in children aged 0–59 months with the largest proportion among children in 12–23 months age category in both pre-vaccine (29.3%) and post-vaccine period (37.2%). Overall, majority of AGE cases were among children below 24 months of age (Table 1).

**Table 1:** Demographic characteristics of the study population in Western and Central Kenya

	Western Kenya *n*=306				Central Kenya *n* = 410			
	Pre-vaccine, *n* = 153		Post-vaccine *n* = 153		Pre-vaccine *n* = 205		Post-vaccine *n* = 205	
	Prevalence	95% C.I	Prevalence	95% C.I	Prevalence	95% C.I	Prevalence	95% C.I
Male	86 (56.3%)	48.3–63.9	91 (59.5%)	51.6–67.0	116 (56.6%)	49.7–63.3	107 (52.2%)	45.4–59.0
Female	67 (43.8%)	36.1–51.8	62 (40.5%)	33.0–48.5	89 (43.4%)	36.7–50.2	98 (47.8%)	41.0–54.6
Age in months								
0–2	0 (0.0%)	0.0–2.0	2 (1.3%)	0.2–4.3	1 (0.5%)	0.0–2.4	4 (2.0%)	0.6–4.6
3–5	17 (11.1%)	6.8–16.6	26 (17.0%)	11.7–23.6	10 (4.9%)	2.5–8.5	15 (7.3%)	4.3–11.5
6–8	24 (15.7%)	10.6–22.1	15 (9.8%)	5.8–15.3	58 (28.3%)	22.5–34.6	43 (21.0%)	15.8–27.0
9–11	15 (9.8%)	5.8–15.3	23 (15.0%)	10.0–21.4	41 (20.0%)	15.0–25.9	26 (12.7%)	8.6–17.8
12–23	45 (29.4%)	22.6–37.0	45 (29.4%)	22.6–37.0	60 (29.3%)	23.4–35.8	76 (37.1%)	30.6–43.9
24–59	52 (34.0%)	26.3–41.8	42 (27.5%)	20.8–34.9	35 (17.1%)	12.4–22.7	41 (20.0%)	15.0–25.9

Pre-vaccine period (Jun 2011–Jan 2014); post-vaccine period (Feb 2014–Oct 2020); Age expressed in months

C.I confidence interval

### Epidemiological trends of Adenovirus, Astrovirus and Norovirus

To determine the epidemiological trends of diarrheal viruses, we compared the prevalence of adenovirus, astrovirus and norovirus before and after the introduction of rotavirus vaccine. Following rotavirus vaccine introduction, we observed a substantial increase in adenovirus in Western Kenya from 9.2% to 12.4%, p = 0.461. Conversely, there was a significant decrease in the same virus in Central Kenya from 17% to 6.3% p = 0.007. Both Western and Central Kenya recorded an insignificant rise in astrovirus and norovirus prevalence following the vaccine introduction (Table 2).

**Table 2:** Prevalence and distribution of enteric viruses in Kenya before and after rotavirus vaccine introduction

Virus	Western Kenya *n* = 306		Central Kenya *n* = 410					
	Pre-vaccine *n* = 153		Post-vaccine *n* = 153		Pre-vaccine *n* = 205		Post-vaccine *n* = 205	
	Prevalence	95% C.I	Prevalence	95% C.I	Prevalence	95% C.I	Prevalence	95% C.I
Adenovirus	14 (9.2%)	5.3–14.5	19 (12.4%)	7.8–18.4	36 (17.6%)	12.8–23.2	13 (6.3%)	3.6–10.3
Astrovirus	5 (3.3%)	1.2–7.1	9 (5.9%)	2.9–10.5	4 (2.0%)	0.6–4.6	5 (2.4%)	0.8–5.3
Norovirus	3 (2.0%)	0.5–5.2	5 (3.3%)	1.2–7.1	11 (5.4%)	2.8–9.1	12 (5.9%)	3.2–9.7

Pre-vaccine period (Jun 2011–Jan 2014); post-vaccine period (Feb 2014–Oct 2020); C.I confidence interval

### Molecular epidemiology of norovirus strains

**Fig. 1:**
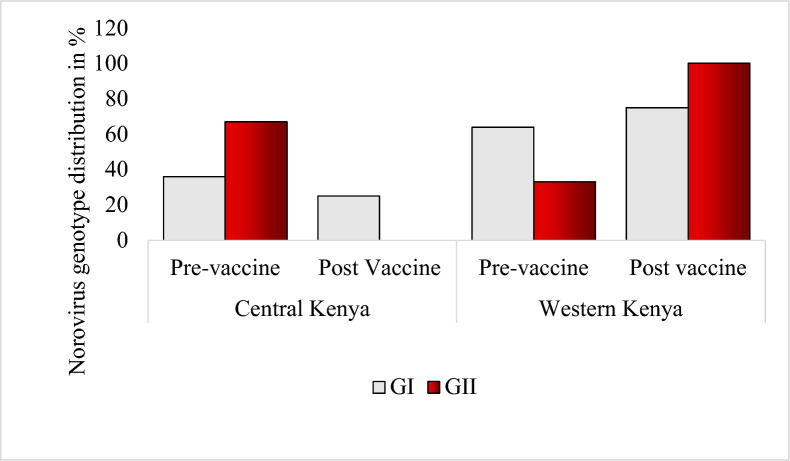
Norovirus genotype distribution in Central and Western Kenya

In Central Kenya, norovirus GII was the dominant strain before and after the introduction of the rotavirus vaccine. In Western Kenya, GI dominated during the pre-vaccine period and declined after the vaccine was introduced (Fig. 1).

### Co-infection with different enteric viruses

A total of 358 fecal samples were analyzed during the pre-vaccine period in both Central and Western Kenya, with no co-infections detected. After the introduction of the rotavirus vaccine, co-infections were observed in 7 out of 358 samples (11.1%). 3 of the 7 co-infections (42.8%) were identified in Central Kenya. Among these, 1 case (33.3%) involved adenovirus and norovirus, while the remaining 2 cases (66.7%) were between norovirus and astrovirus. 4 of the 7 co-infections (57.1%) were observed in Western Kenya. Of these, 50% were between adenovirus and astrovirus, and the other 50% were between adenovirus and norovirus.

### Age distribution of enteric viruses in Central and Western Kenya

**Fig. 2:**
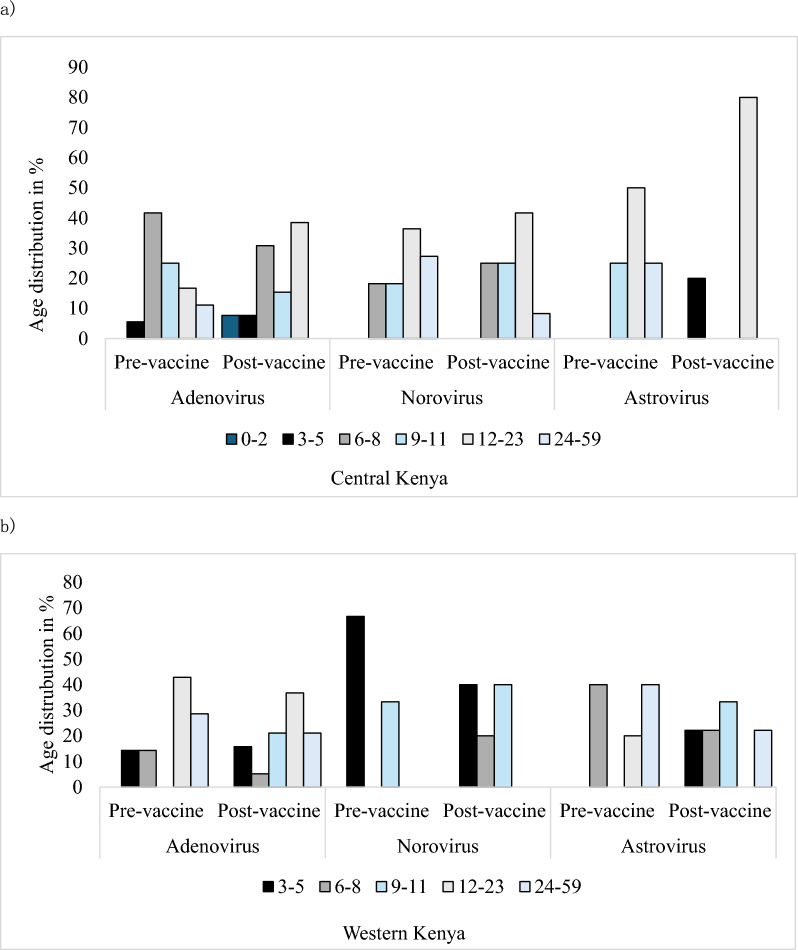
Age distribution of enteric viruses in **a** Central and **b** Western Kenya before and after rotavirus vaccine introduction

Before the introduction of the rotavirus vaccine, adenovirus cases peaked at 6–8 (41.7%) months in Central Kenya and 12–23 (42.9%) months in Western Kenya. After the vaccine, adenovirus cases were most common among children aged 12–23 months in both Central (38.5%) and Western (36.8%) Kenya. Norovirus prevalence peaked at 12–23 (36.4%) months in Central Kenya and 3–5 (41.7%) months in Western Kenya in the pre-vaccine period. Following the vaccine introduction, norovirus was most detected among children aged 12–23 months in Central and 3–5 and 9–11 (40%) months in Western Kenya. Astrovirus infections in Central Kenya were highest among the 12–23 months age group before (50%) and after (100%) the vaccine introduction. In Western Kenya, the highest pre-vaccine prevalence shifted from 6–8 and 24–59 months (40%) in the pre-vaccine period to 9–11 (33.3%) months in the post-vaccine era. (Fig. 2a (Central Kenya) and Fig. 2b (Western Kenya)).

### Rotavirus vaccine coverage among children infected with enteric viruses in Central and Western Kenya

In Central Kenya, good rotavirus vaccination coverage was observed among children, with 100% of them having received full vaccination. Similarly, in Western Kenya, the vaccination coverage was notably high, with over 80% of children having completed their vaccination series, while less than 20% remained uncertain about their vaccination status (Table 3).

**Table 3:** Vaccination status for positive cases during the post-vaccine era in both Central and Western Kenya

Vaccination status	Central Kenya			Western Kenya		
	Adenovirus (%)	Astrovirus (%)	Norovirus (%)	Adenovirus (%)	Astrovirus (%)	Norovirus (%)
Vaccinated	100.0	100.0	100.0	89.5	66.7	100.0
Not vaccinated	0.0	0.0	0.0	0.0	0.0	0.0
ASKU	0.0	0.0	0.0	10.5	33.3	0.0

ASKU, Asked but Unknown

The guardian could neither confirm if the child was vaccinated or not and they also didn’t have a card to show

### Clinical severity among children infected with enteric viruses in Central and Western Kenya

**Fig. 3:**
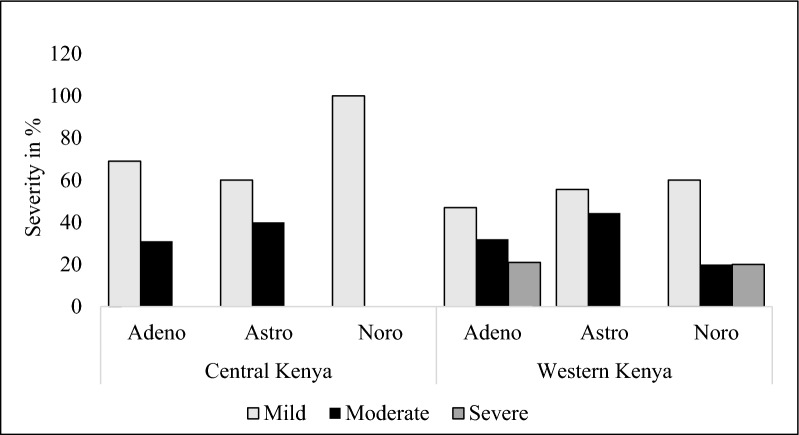
Clinical severity among diarrheal children in Western and Central Kenya after rotavirus vaccine introduction

To assess the diarrheal disease severity among the sample population, the 20-point Vesikari Clinical Severity Scoring System was used. Adenovirus infections were mildly distributed in both Central (69%) and Western Kenya (47%). Astrovirus mild infections were predominant in both Central (60%) and Western Kenya (55%). All the norovirus infections were mild in Central Kenya while 60% of norovirus infections were mild in Western Kenya. We did not have severe infections in Central Kenya, although adenovirus and norovirus reported severe disease in Western Kenya at 21% and 20%, respectively. We did not have clinical severity data during the pre-vaccine era (Fig. 3).

## Discussion

The national roll-out of the rotavirus vaccine in Kenya in July 2014 has provided an opportunity to assess the real-world impact of the vaccine on diarrheal disease burden and molecular epidemiology of rotavirus and other diarrheal viruses such as adenovirus, astrovirus and norovirus in the country. While rotavirus vaccines have shown effectiveness through reduced hospitalizations for all-cause AGE globally [24] and decreased prevalence of rotavirus infection in Kenya [11, 25, 26] and Africa [27] where AGE is a major burden [2],-there is paucity of data on the impact of these vaccines on norovirus, adenovirus 40/41, and astrovirus diarrheal disease burden, particularly in Kenya and other developing countries. In view of this, we sought to determine the disease burden and molecular epidemiology of adenovirus, astrovirus and norovirus in Central and Western Kenya before and after the rotavirus vaccine introduction.

Our data revealed that adenovirus had the highest prevalence during the study period in both Central and Western Kenya compared to other viruses. There was a significant decrease in adenovirus detection in Central Kenya following rotavirus vaccine introduction from 17.6% (95% CI 12.8–23.2) to 6.3% (95%CI 3.6–10.3) and a non-significant increase from 9% (95% CI 5.3–14.5) to 12.4% (95%CI 7.8–18.4) in Western Kenya. The findings in Western Kenya were consistent with a study done in Kenya at Kilifi County, which reported both rotavirus and non-rotavirus gastroenteritis [1]. The decline for adenovirus in Central Kenya is not clear and underscores the need for continued surveillance with a higher number of samples. There was a non-significant increase in astrovirus and norovirus following vaccine introduction in both geographical regions, with norovirus findings similar to a study conducted in US [6] and Bolivia [28]. Norovirus GII increased post-vaccine introduction in both Central and Western Kenya, our findings are consistent with a study done in Kenya [1] and Nicaragua [29].

Children aged 12–24 months recorded the highest prevalence among the three viruses in Central Kenya both before and after the rotavirus vaccine introduction. This data conforms to the reports on a study conducted in the USA in 2021 which noted a prevalence among the 18–23 age group [30]. In Western Kenya, there was a shift in adenovirus infections from 6–8 months to 12–23 months following vaccine introduction. Astrovirus and norovirus infections were more prevalent in children aged below 1 year in both pre-vaccine and post-vaccine periods. Rotavirus vaccines are administered at 6–12 weeks of age [31] and are highly effective in preventing rotavirus infection, but their impact on immunity to other enteric viruses remains under investigation.

In this study, both geographical regions had good vaccine coverage > 80% showing good access to vaccines. We used a 20-point clinical severity scoring system to assess the severity of entero-pathogens diarrheal positive cases post-vaccine introduction. In Central Kenya, norovirus recorded mild cases while adenovirus and astrovirus recorded mild to moderate cases. In Western Kenya, adenovirus and norovirus recorded mild-moderate to severe cases while astrovirus had mild-moderate cases. The Rotavirus vaccine is effective in reducing the severity of rotavirus diarrhea [32]. However, we lacked baseline data to show its impact on the clinical severity of other enteric viruses.

Our study was limited to using rotavirus-negative samples, for which there is limited literature available for direct comparison in this cohort. Additionally, the study included only inpatients during the pre-vaccine period, while the post-vaccine period comprised both inpatients and outpatients, making direct comparisons challenging. Furthermore, the sample size was small, and data collection did not cover all seasons, preventing an analysis of the seasonality of enteric viruses.

## Conclusion

To the best of our knowledge, this is the first study to assess the prevalence of norovirus, astrovirus, and adenovirus in children with AGE who tested negative for rotavirus. Our data highlights the burden of norovirus, astrovirus and adenovirus among children presenting with acute gastroenteritis and are negative for rotavirus in Central and Western Kenya. These findings underscore the need for continued monitoring for implementation of appropriate public health measures.

## Data Availability

No datasets were generated or analysed during the current study.
